# Supplementary material for: CD169+ macrophages orchestrate plasmacytoid dendritic cell arrest and retention for optimal priming in the bone marrow of malaria-infected mice
Source: eLife. 2022 Oct 24;11:e78873. doi: 10.7554/eLife.78873 (PMC9648966; doi:10.7554/eLife.78873)
Supplement: Supplementary file 1. [file elife-78873-supp1.pdf]

Reagent/Antibody	Fluorochrome	Clone	Purchased from
**FACS**			
anti-BST2	APC, PE	eBio129c	eBioscience
anti-CD3	PerCPCy5.5, BV510	*145-2C11 or* 17A2	Biolegend, BD Biosciences
anti-CD19	PerCpcy5.5, BV510	1D3	eBioscience, BD Biosciences
anti-CX3CR1	PE-Cy7	SA011F11	Biolegend, BD Biosciences
anti-NK1.1	PerCpcy5.5, BV510	PK136	eBioscience, BD Biosciences
anti-Ly-6C	PerCpcy5.5, FITC	HK1. 4 AL-21	BD Biosciences
anti-CD11a	Biotin, APC	M17/4	eBioscience
anti-CD11b	eF450, BB700, FITC	M1/70	BD Biosciences
anti-CD169	PE, APC, FITC	SER4	eBioscience
anti-Ly-6G	AF700, PE	1A8	BD Biosciences
anti-F4/80	BV421	BM8	Biolegend, eBioscience
anti-Gr1	BV510	RB6-8C5	BD Biosciences
anti-CD45	Alexa 700	30-F11	BD Biosciences
anti-CD80	PE-Cy7	16-10A1 1615A1	eBioscience, BD Biosciences
anti-CD86	PE, APC	GL1	eBioscience, BD Biosciences
anti-MHCII I-Ab	FITC	AF6-120.1 M5/114.15.2	eBioscience
anti-ICAM-1	Biotin, PE	YN1.1.7.4, 3E2	eBioscience, BD Biosciences
anti-VCAM1	PE-Cy7	429	eBioscience
CD62-L	AF700	MEL-14	Biolegend
anti-SiglecH	PE-Cy7, AF647	eBio440c	eBioscience
anti-Sca-1	Biotin, Alexa 700	D7	eBioscience
anti-CD49d	PE	R1-2	eBioscience
anti-CD115	PE-Cy7	AFS98	eBioscience
anti-CXCR3	PerCpcy5.5	CXCR3-173	eBioscience
anti-CXCR4	PerCpcy5.5 eFluor710	2B11	eBioscience
anti-CCR9	Pe-Cy7	eBiocw-1.2	eBioscience
LIVE/DEAD	Ghostred780		Tonbo
streptavidin	eFluor710, PerCpcy5.5		eBioscience
anti-Ter119	Biotin	TER-119	eBioscience
**Intravital Microscopy**			
anti-CD169	PE	SER4	eBioscience
***In vivo* depletion**			
anti-CXCR3		CD183	BioXcell
anti-LFA-1α (CD11a)		M17/4	BioXcell
anti-VLA-4 (CD49d)		PS/2	BioXCell
CXCL9		2A6.9.9	Andrew Luster, Robert Schreiber
Rat IgG2b, κ			BioXCell
Rat IgG2a, Rat IgG2b, κ			BioXCell
Armenian Hamster IgG			BioXCell

Table S1
